# Margination and stretching of von Willebrand factor in the blood stream enable adhesion

**DOI:** 10.1038/s41598-017-14346-4

**Published:** 2017-10-27

**Authors:** Kathrin Rack, Volker Huck, Masoud Hoore, Dmitry A. Fedosov, Stefan W. Schneider, Gerhard Gompper

**Affiliations:** 1 Theoretical Soft Matter and Biophysics, Institute of Complex Systems and Institute for Advanced Simulation, Forschungszentrum Jülich, 52425 Jülich, Germany; 2 Simulation and Software Technology, German Aerospace Center, 51147 Köln, Germany; 3 University Medical Center Hamburg-Eppendorf, Center for Internal Medicine, 20246 Hamburg, Germany; 4 Heidelberg University, Medical Faculty Mannheim, Experimental Dermatology, 68167 Mannheim, Germany

## Abstract

The protein von Willebrand factor (VWF) is essential in primary hemostasis, as it mediates platelet adhesion to vessel walls. VWF retains its compact (globule-like) shape in equilibrium due to internal molecular associations, but is able to stretch when a high enough shear stress is applied. Even though the shear-flow sensitivity of VWF conformation is well accepted, the behavior of VWF under realistic blood flow conditions remains poorly understood. We perform mesoscopic numerical simulations together with microfluidic experiments in order to characterize VWF behavior in blood flow for a wide range of flow-rate and hematocrit conditions. In particular, our results demonstrate that the compact shape of VWF is important for its migration (or margination) toward vessel walls and that VWF stretches primarily in a near-wall region in blood flow making its adhesion possible. Our results show that VWF is a highly optimized protein in terms of its size and internal associations which are necessary to achieve its vital function. A better understanding of the relevant mechanisms for VWF behavior in microcirculation provides a further step toward the elucidation of the role of mutations in various VWF-related diseases.

## Introduction

The blood glycoprotein von Willebrand factor (VWF) is involved in hemostasis, as it mediates platelet adhesion at vessel walls in case of an injury^1–3^. VWF can bind flowing platelets and withstand very high shear forces^4^, an essential aid at high shear rates where platelets cannot bind autonomously^5,6^. Multimeric VWF is the largest protein in blood, with a contour length reaching up to 250 μm^1,7^. VWF is stored at and released from endothelial cells and is present freely floating (called plasmatic VWF) in blood. The release of stored VWF can be triggered by the signals of a vascular damage or in certain blood diseases^2,8^. Stored VWF is often much longer than plasmatic VWF, since the length of plasmatic VWF is controlled by the ADAMTS13 protease, which is able to cut a VWF-chain if a cleavage site is exposed^9–11^. Various VWF dysfunctions can lead to uncontrolled bleeding as in the von Willebrand disease (VWD)^12–14^ or to spontaneous thrombotic events^12^. In addition to the pivotal role of VWF in hemostasis, there is a growing evidence that VWF plays an important role in cancer^15,16^.

Several experimental studies^17–19^ have focused on adhesion of platelets to VWF, suggesting that the adhesion depends strongly on the shear rate and the length of VWF. These studies also indicate that with increasing shear rate, a conformational change of VWF occurs both at a dimeric level (opening-up of the dimer structure)^1,20^ and multimeric level (stretching of VWF from a compact globule-like form to an extended chain form)^21^, and is accompanied by an increased platelet adhesion. Recent experiments^21^ and simulations^22^ for a single VWF in shear flow have shown that the extension of VWF is shear-rate dependent and that VWF stretches above a critical shear rate of several thousand s^−1^ ^21^. A mixture of VWF and platelets has been found to exhibit the formation of reversible aggregates at high shear rates, which disappear when the shear rate is decreased^23,24^. However, there is still no comprehensive picture of VWF behavior in the microcirculation, where various flow rates and hematocrit values *H*_*t*_ (the volume fraction of red blood cells (RBC)) are encountered.

The distribution and stretching of VWF under realistic blood-flow conditions are important for understanding its behavior in the microvasculature. Several experimental^25,26^ and numerical^27,28^ studies have shown that flexible polymers in a dilute suspension are subject to cross-stream migration in Couette and Poiseuille flow. Furthermore, platelets^29^ and micro-particles^30,31^ are known to marginate (i.e., migrate toward vessel walls) in blood flow. Margination of spherical micron-size particles is mediated by RBCs, which migrate to the center of a vessel due to both hydrodynamic interactions with the walls (also called lift force)^32–34^ and asymmetric collisions of non-spherical and deformable objects under flow^35^ leading to a RBC-free layer (RBC-FL) near the walls^36^ and expelling the particles into the RBC-FL. Particle deformation generally results in less margination in blood flow^37^. Thus, a detailed understanding of the margination and extension properties of large VWF multimers under realistic microcirculatory conditions is required for the interpretation of their adhesive behavior.

In this paper, we study margination and stretching of large multimeric VWFs for a wide range of flow rates and *H*_*t*_ values using mesoscopic hydrodynamic simulations, and consistently link the obtained picture of VWF behavior in blood flow to our microfluidic experiments on VWF margination and adhesion. We find that large multimeric VWFs mainly retain their compact shape within the blood-flow core populated primarily by RBCs, and that this compact configuration facilitates their efficient margination into the RBC-FL. After margination, VWF stretches in the RBC-FL near a wall, thereby facilitating the adhesion to the vessel wall. VWF margination and extension have a non-trivial dependence on flow rate, *H*_*t*_, and the contour length of VWF. The proposed dynamics of VWF in blood flow obtained from simulations is consistent with our experimental observations and provides an excellent explanation for VWF adhesion with respect to various investigated conditions. Our findings are also relevant for VWF-pertinent blood diseases resulting from various VWF mutations.

## Results

### Simulation and experimental measurements

**Figure 1:**
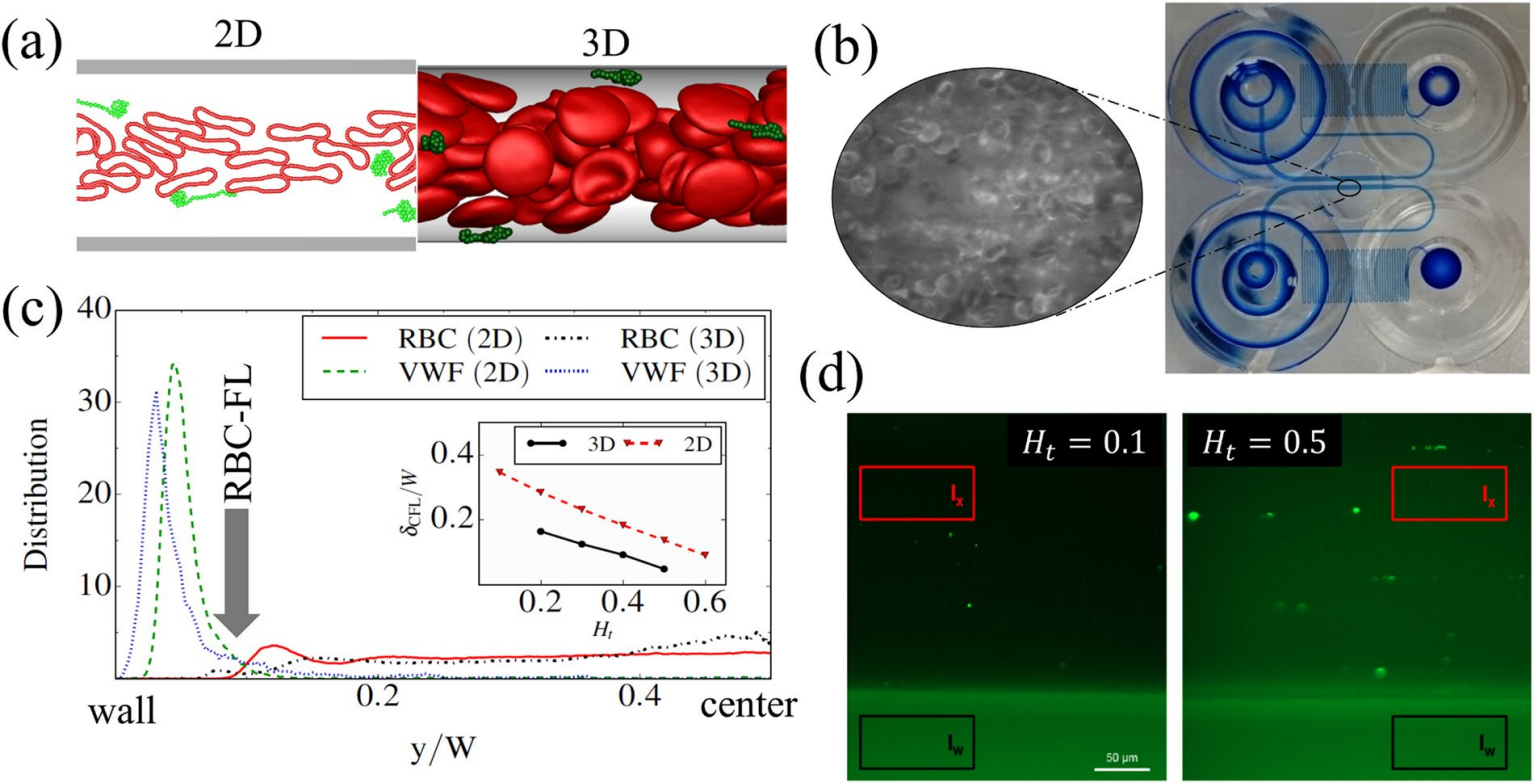
Simulation and experimental measurements. (**a**) Snapshots from 2D and 3D simulations with RBCs colored in red and VWFs in green. The simulation setup corresponds to a cylindrical microvessel in 3D with the diameter *W* = 20 μm or to a channel in 2D with the width *W* = 20 μm. (**b**) Experimental setup consists of a microfluidic system with multiple identical channels (two channels are shown). At the pre-processing step, channel walls are coated with collagen. Blood with different *H*_*t*_ levels is perfused through the device (from an inlet on the right to an outlet on the left) using a pneumatic pump. Optical microscopy is performed in the middle of the wide-channel section marked by the small oval area, where we can observe RBCs but not VWF because it needs to be detected by fluorescence microscopy. (**c**) Simulation measurements supply distributions of different components in blood flow. The plot shows a comparison of center-of-mass (COM) distributions of RBCs and VWFs obtained from a 2D simulation with *H*_*t*_ = 0.6 and a 3D simulation with *H*_*t*_ = 0.4 for a nearly same thickness of RBC-FL and a pseudo-shear rate of ${\dot{\gamma }}^{\ast }\approx 60$ ($\bar{\dot{\gamma }}\approx 62$ s^−1^). The distributions from 2D and 3D simulations compare well for a fixed thickness of the RBC-FL, indicating that hematocrit values in 2D correspond to lower hematocrits in 3D. The inset presents the RBC-FL thicknesses δ_CFL_ for different *H*_*t*_ and demonstrates that a negative shift by about 0.15–0.2 is required to relate *H*_*t*_ in 2D to that in 3D. (**d**) Experimental measurements provide fluorescence intensity of labelled VWFs near the channel bottom. The isotropic background level of measured intensity (*I*_x_) characterizes the amount of flowing VWF near the wall (or VWF margination), while the bright spots correspond to adhered VWF molecules or aggregates. The intensity in the right plot for *H*_*t*_ = 0.5 is clearly stronger than on the left for *H*_*t*_ = 0.1 indicating that VWF margination is more pronounced at *H*_*t*_ = 0.5. All measured intensities are normalized by the wall autofluorescence intensity *I*_w_ (the black marked area), which remains constant throughout all performed experiments. Scale bars correspond to 50 μm.

Blood is modeled as a suspension of RBCs^38^ and a few macromolecules (e.g., flexible polymers, VWFs), which flow in a cylindrical microvessel in 3D with diameter *W*, or in a channel in 2D with width *W*, see Fig. 1(a) and *Methods* for technical details. A VWF model has to incorporate internal associations within a molecule, which give rise to its compact shape in the absence of flow, and reproduce the stretching of VWF in shear flow above a critical shear rate^21^. The internal associations can be modelled explicitly by intra-molecular dynamic bonds, as in a self-associating polymer model^39–41^, or implicitly by imposing pairwise attractive interactions between the monomeric units of a polymer^22,42,43^. We have selected the VWF model with inter-bead attractive interactions^22,42,43^ for our simulations, since it is able to reproduce quantitatively the experimentally observed stretching of VWF in shear flow^21^. The resultant potential between polymer units is the superposition of a harmonic bond potential and an attractive Lennard-Jones (LJ) pairwise potential. The VWF model includes the attractive part of LJ interaction while the repulsive polymer model (e.g., self-avoiding polymer) excludes the attractive part of the LJ potential. The repulsive polymer model is employed for comparison with the VWF model in order to demonstrate the importance of VWF self-associations and/or the significance of VWF critical stretching for its behavior in blood flow. Calibration of the attractive LJ interactions for the VWF model against the experimental data of VWF stretching in simple shear flow^21^ is displayed in Supplementary Fig. S1.

The flow strength is characterized by a non-dimensional shear rate defined as ${\dot{\gamma }}^{\ast }=\bar{\dot{\gamma }}{\tau }_{{\rm{RBC}}}=\bar{\dot{\gamma }}\eta {D}_{r}^{3}/{\kappa }_{r}$, where $\bar{\dot{\gamma }}=\bar{v}/W$ is the average shear rate (or pseudo shear rate) with average flow velocity $\bar{v}$ computed from the velocity profile, and τ_RBC_ is the characteristic RBC relaxation time defined through the fluid viscosity η, effective RBC diameter *D*_*r*_, and the membrane bending rigidity ${\kappa }_{r}$. Here, ${D}_{r}=\sqrt{{A}_{0}/\pi }$ in 3D and ${D}_{r}={L}_{0}/\pi$ in 2D, with *A*_0_ being the RBC surface area in 3D and *L*_0_ the cell circumference in 2D. The characteristic relaxation time is ${\tau }_{{\rm{RBC}}}\approx \mathrm{1.1\ }{\rm{s}}$ for healthy RBCs under physiological conditions^31,44^, and therefore, the pseudo-shear rate $\bar{\dot{\gamma }}$ is roughly equivalent in magnitude to ${\dot{\gamma }}^{\ast }$ in inverse seconds.

The experimental setup corresponds to a microfluidic system with multiple identical channels of a rectangular cross-section, see Fig. 1(b) and *Methods* for details. The walls of the channel are functionalized with collagen to facilitate the adhesion of VWF, while the flow in the channel is controlled by an electropneumatic pump. Controlled parameter in experiments is the wall shear rate ${\dot{\gamma }}_{{\rm{w}}}$, which can be related to the pseudo shear rate $\bar{\dot{\gamma }}$ as ${\dot{\gamma }}_{{\rm{w}}}\ge 8\bar{\dot{\gamma }}$ for a tube flow in 3D (${\dot{\gamma }}_{{\rm{w}}}\ge 6\bar{\dot{\gamma }}$ for a channel in 2D), where the equal sign holds only for a parabolic flow with a Newtonian fluid (e.g., only blood plasma). The presence of RBCs can make the ratio ${\dot{\gamma }}_{{\rm{w}}}/\bar{\dot{\gamma }}$ significantly larger than 8 in 3D (or 6 in 2D), especially for high hematocrits (*H*_*t*_ ≥ 0.3), as illustrated in Supplementary Fig. S2.

Simulations constitute the typical distributions of blood components for RBCs and VWF, as shown in Fig. 1(c) in 2D with *H*_*t*_ = 0.6 and in 3D with *H*_*t*_ = 0.4 for a nearly same thickness of the RBC-FL and a pseudo-shear rate of ${\dot{\gamma }}^{\ast }\approx 60$ ($\bar{\dot{\gamma }}\approx 62$ s^−1^). The distributions of VWF clearly show a peak near the channel wall indicating strong margination of the VWF polymers, while RBCs mainly populate the center of the channel. The thickness of the RBC-FL is calculated based on the edge of RBC core of the flow, analogously to the measurements of RBC-FL in refs^36,45^. It is important to note that the 2D and 3D simulations are compared at different *H*_*t*_ values but for a nearly same thickness of RBC-FL, since margination of particles in blood flow is strongly correlated with the RBC-FL thickness^37,46^. The good correspondence of the distributions for a fixed RBC-FL thickness demonstrates that *H*_*t*_ value in 2D has a different meaning than in 3D. The inset in Fig. 1(c) presents the RBC-FL thicknesses *δ*_CFL_ for different *H*_*t*_ values, indicating that a negative shift by about 0.15–0.2 is required to relate *H*_*t*_ in 2D to that in 3D.

Throughout the paper, we will mainly employ 2D simulations to enable a systematic investigation of the behavior of VWF in blood flow for a wide range of conditions (e.g., hematocrit, flow rate, VWF size), which would be computationally intractable in 3D. However, the 2D model qualitatively reproduces the relevant physical effects in blood flow in comparison to 3D, as demonstrated in Fig. 1(c). Furthermore, we have verified that 2D and 3D simulations agree well at several selected *H*_*t*_ values. The similarity between 2D and 3D simulations has also been shown for the margination of micro- and nano-carriers in blood flow^31^.

In experiments, blood samples with different hematocrits are perfused through the channel in order to monitor margination and adhesion of fluorescently-labeled VWF. VWF margination is measured through isotropic background level of the fluorescence intensity *I*_x_ near the bottom wall of the channel, as shown in Fig. 1(d). *I*_x_ is normalized by the autofluorescence intensity *I*_w_ of the lateral channel wall, which remains constant throughout all performed experiments. VWF adhesion is quantified by the intensity and area of the immobile bright spots illustrated in Fig. 1(d), which correspond to adhered VWF molecules or their aggregates. A detailed measurement of VWF distribution along the channel cross-section, as it is done in simulations, is not possible experimentally since individual VWF chains cannot be observed and tracked. For the same reason, it is not feasible to identify the composition of immobile aggregates adhered at the channel wall. Finally, the distribution of VWF lengths in experiments corresponds to an average physiological distribution in blood, which is clearly polydisperse, while simulations are performed for a fixed length of VWF. Therefore, a direct comparison between simulations and experiments is not intended; however, we consistently integrate our numerical and experimental observations in order to understand better VWF behavior in blood flow.

### VWF margination and stretching in blood flow

Margination properties of large VWFs in blood flow are very important for their efficient adhesion, since margination is a necessary precondition for adhesion. In order to quantify VWF margination properties, we define margination probability *P* as a probability of the polymer’s center of mass (COM) to be within a certain distance *δ* away from the wall. Throughout the paper, *δ* = 2 μm is chosen for the calculation of VWF margination, motivated by the range of potential VWF adhesion. Note that this choice only affects the magnitudes of margination probabilities, but their relative importance for different investigated conditions remains unaffected.

**Figure 2:**
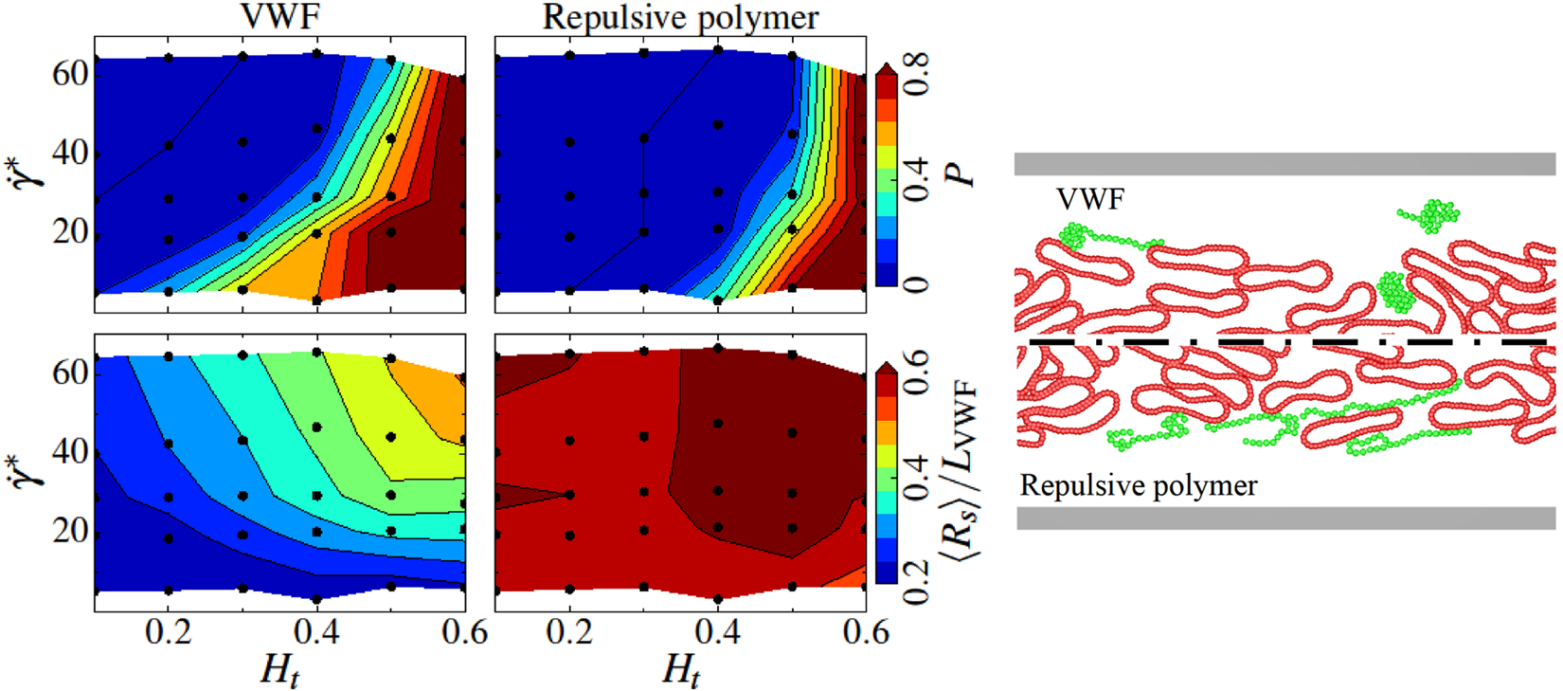
Simulated margination and stretching of VWF and a repulsive polymer. Comparison of margination and stretching of VWF (left column) and a repulsive polymer (right column) in blood flow. On the right, the simulation snapshots are presented for the conditions *H*_*t*_ = 0.3 and ${\dot{\gamma }}^{\ast }\approx 29.7$ ($\overline{\dot{{\rm{\gamma }}}}\approx 31\,{{\rm{s}}}^{-1}$), where RBCs are colored in red while VWFs and repulsive polymers are in green. Clearly, the repulsive polymer exhibits a much stronger extension in blood flow in comparison to VWF. The upper row shows margination probability *P* into the near-wall layer with a 2 μm thickness for VWF and a repulsive polymer as a function of hematocrit *H*_*t*_ and shear rate ${\dot{\gamma }}^{\ast }$. Both the VWF and repulsive polymer consist of 26 beads corresponding to the contour length of *L*_VWF_ = 15 μm. The black dots indicate the values of *H*_*t*_ and ${\dot{\gamma }}^{\ast }$ for which simulations have been performed. The color code ranges from blue (low probability) to red (high probability) and is acquired via interpolation. The bottom row compares the normalized average stretching <*R*_*s*_>/*L*_VWF_ of VWF and the repulsive polymer within the RBC-FL.

Simulations performed for a wide range of *H*_*t*_ and ${\dot{\gamma }}^{\ast }$ values allow the construction of a margination diagram of VWF in blood flow (Fig. 2 (top,left)) which clearly identifies the margination properties of VWF. Margination becomes stronger with increasing *H*_*t*_ because RBCs mediate this process by migrating to the vessel center and expelling VWF toward vessel walls. Interestingly, VWF margination is not enhanced with increasing ${\dot{\gamma }}^{\ast }$ (or flow rate), which is due to the deformation and extension of VWF in flow. At high shear rates, a VWF molecule is subject to significant elongation in flow (i.e., VWF resembles an ellipsoidal shape), which results in a lift force pushing a deformed molecule away from the walls. Indeed, recent works about the lift force on polymers^47^ and vesicles^32–34^ near surfaces in simple shear flow suggest that the lift force strongly increases with a polymer or vesicle size. The lift force generally arises from hydrodynamic interactions between deformable objects and a wall in flow^32–34^ which is, inter alia, a primary reason for the migration of RBCs toward the vessel center. Thus, the distribution of VWF within a vessel cross-section in microcirculation strongly depends on local blood-flow conditions.

In addition to the margination of VWF, another important aspect for VWF adhesion is its extension in flow, since in a compact configuration internal adhesive sites are shielded and VWF remains non-adhesive. Stretching of large VWFs is shown in Fig. 2 (bottom,left) for various conditions. These data correspond to an average extension $< {R}_{S} >$ in the flow direction within the RBC-FL, where the shear rate is largest. Clearly, VWF stretches more with increasing non-dimensional shear rate ${\dot{\gamma }}^{\ast }$ or equivalently wall shear rate ${\dot{\gamma }}_{{\rm{w}}}$. The dependence on *H*_*t*_ also indicates a stronger extension for larger hematocrits. The latter dependence arises from the effect that VWF is effectively confined in the RBC-FL between the wall and the RBC core. The thickness of RBC-FL decreases with increasing *H*_*t*_ as shown in the inset of Fig. 1(c). This behavior is not entirely surprising and is consistent with the previous results showing a stronger extension of polymers and VWF in close proximity to the wall than in bulk flow^21,43^. In addition, recent simulations^48^ suggest that the collision of VWF with colloidal particles can significantly enhance its stretching, which may lead to VWF stretching in the bulk of the flow. In our simulations, we do not observe much stretching of VWF within the RBC-core of the flow, mainly due to the relatively low shear rates in the bulk. The largest wall-shear rates in our simulations are below 700 *s* ^−1^ and shear rates in the bulk are much smaller than this value. Clearly, at much higher flow rates than those investigated here, significant stretching of VWF in the RBC core should be also expected.

Figure 2 also presents analogous results for a repulsive self-avoiding polymer (i.e., a polymer without attractive interactions between monomers). A striking difference is that margination of a repulsive polymer is substantially less pronounced in comparison to VWF. Two mechanisms can be identified, which are both closely related to the higher ease of deformation and stretching of a repulsive polymer in blood flow (see Fig. 2(bottom,right)). In the vicinity of the wall, the resulting elongated shape implies a larger lift force^47^, which pushes the polymer back into the RBC core. Inside the core, collisions of soft (polymer) and stiff (RBC) objects lead to a displacement of the softer object toward the channel center^35^; however, this effect weakens for very soft objects, as they can “sneak” around stiffer cells more easily. In contrast, VWF remains mainly in a compact configuration within the RBC flow core and displays some degree of stretching primarily in the RBC-FL, see the snapshots in Fig. 2. This means that a compact configuration of VWF does not only prevent undesired adhesion (as adhesive sites remain hidden), but also facilitates an efficient margination in blood flow. Another striking difference between VWF and the repulsive polymer is that stretching of the repulsive polymer near the wall is much stronger than that of VWF and is nearly independent of the shear rate and hematocrit, as can be seen from the stretching diagram in Fig. 2(bottom,right).

**Figure 3:**
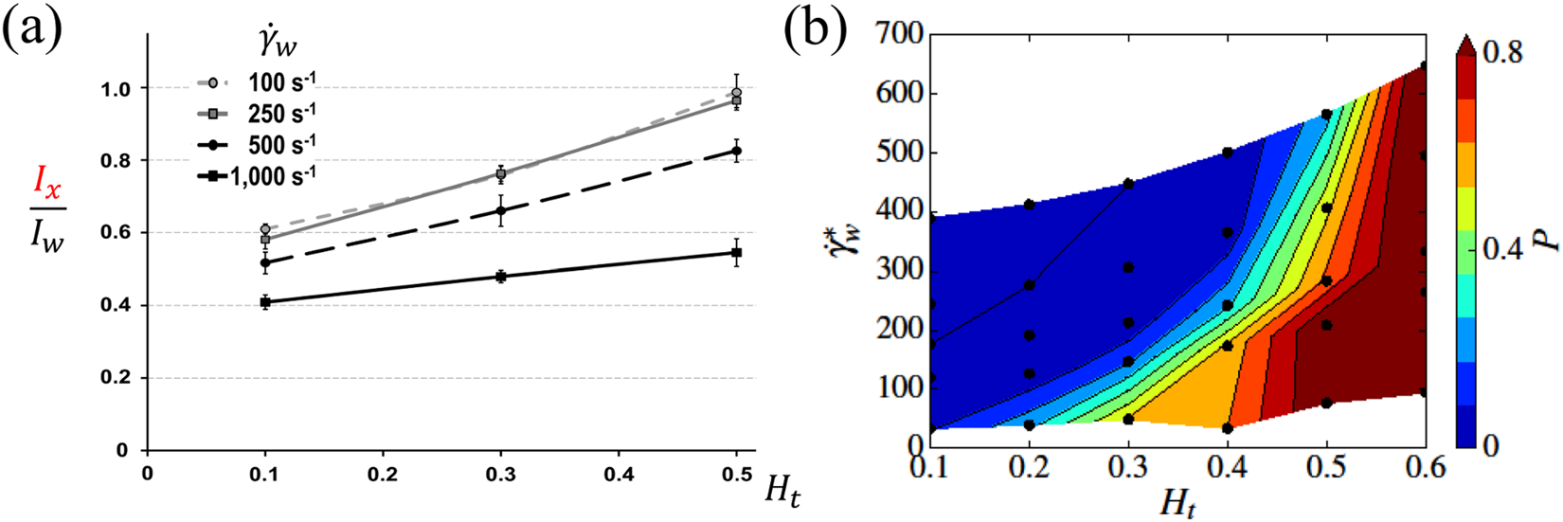
Margination of VWF in microfluidics and simulations. (**a**) Experimental data of VWF margination as a function of hematocrit and wall shear rate ${\dot{\gamma }}_{{\rm{w}}}$. For every point, at least four independent experiments were made. (**b**) Simulation results for VWF margination into a 2 μm layer near the wall as a function of *H*_*t*_ and the normalized wall shear rate. The data in the diagram are same as those in Fig. 2(top,left), but the VWF margination probability is plotted as a function of ${\dot{\gamma }}_{{\rm{w}}}^{\ast }={\dot{\gamma }}_{{\rm{w}}}{\tau }_{{\rm{RBC}}}$ (${\tau }_{{\rm{RBC}}}\approx \mathrm{1.1\ }{\rm{s}}$) instead of ${\dot{\gamma }}^{\ast }$ for a better comparison between simulations and experiments. Note that only a qualitative comparison of margination is intended here.

The experimental margination data are presented in Fig. 3(a) for different wall shear rates ${\dot{\gamma }}_{{\rm{w}}}$, which can be compared qualitatively to corresponding simulation results in Fig. 3(b). Experiments and simulations (remember a shift in *H*_*t*_ in the relation between 2D and 3D) consistently yield an increase of margination with increasing *H*_*t*_ and a decrease in margination with increasing wall shear rate. A quantitative comparison between experiments and simulations is not possible here, since experimental samples contain a distribution of different VWF lengths and separate VWF molecules cannot be distinguished, as discussed above. However, experimental and simulation results show a good qualitative agreement for the dependence of VWF margination on *H*_*t*_ and shear rate.

**Figure 4:**
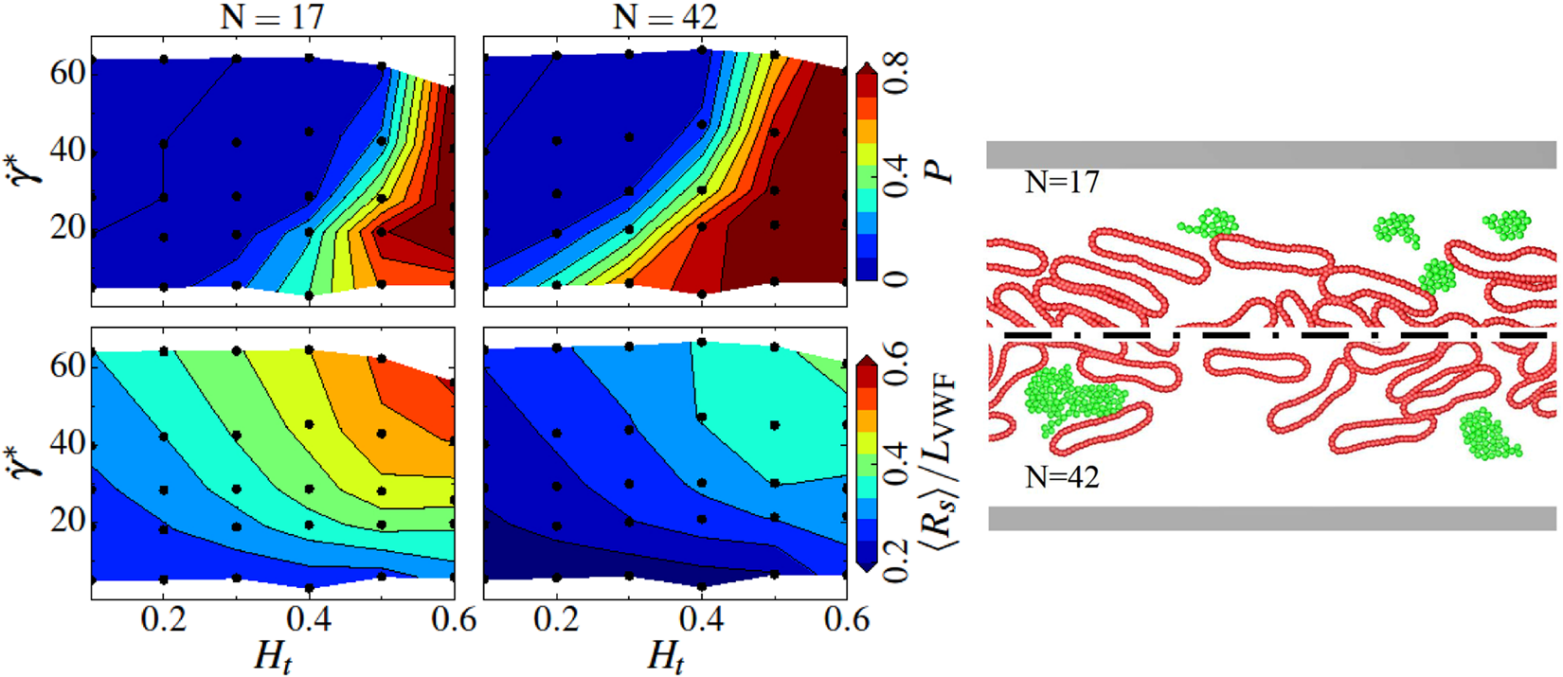
Simulated margination and stretching of VWFs with different lengths. Comparison of margination and stretching of VWFs with a different number of monomers: left column - *N* = 17 (*L*_VWF_ = 9.6 μm) and right column - *N* = 42 (*L*_VWF_ = 24.6 μm). On the right, simulation snapshots are presented for the conditions *H*_*t*_ = 0.3 and ${\dot{\gamma }}^{\ast }\approx 29.7$ ($\bar{\dot{\gamma }}\approx 31$ s^−1^), where RBCs are colored in red while VWFs are in green. The upper row shows margination probability *P* into the near-wall layer with a 2 μm thickness for VWF with a different length as a function of hematocrit *H*_*t*_ and shear rate ${\dot{\gamma }}^{\ast }$. The black dots correspond to the values of *H*_*t*_ and ${\dot{\gamma }}^{\ast }$ for which simulations have been performed. The color code ranges from blue (low probability) to red (high probability) and is acquired via interpolation. The bottom row compares the normalized average stretching <*R*_*s*_>/*L*_VWF_ of VWFs within the RBC-FL.

Simulation results for the dependence of VWF margination on its length are presented in Fig. 4 for VWF models with *N* = 17 and *N* = 42 beads, corresponding to *L*_VWF_ = 9.6 μm and *L*_VWF_ = 24.6 μm, respectively, which should be also compared with Fig. 2 for *N* = 26 (*L*_VWF_ = 15 μm). The comparison indicates that smaller VWF molecules are marginated less efficiently. This observation is consistent with the fact that micron-sized particles possess better margination properties than their sub-micron counterparts^31^. The large VWF with *N* = 42 shows similar margination properties as the VWF with *N* = 26 in Fig. 2. Thus, the most efficient VWF margination is expected to occur for large VWF molecules with a contour length greater than about 12–13 μm.

An interesting observation from Fig. 4(bottom) is that a shorter VWF stretches generally better than longer chains. A similar behavior has been predicted by the theoretical analysis of shear-induced unfolding of self-attracting polymeric globules^22^, where the critical shear rate for unfolding has been found to depend linearly on the characteristic radius of the globule. Thus, the critical shear rate for VWF stretching is expected to have a weak dependence on *N* ($\approx {N}^{\mathrm{1/3}}$) and shorter VWFs are able to stretch at lower shear rates than longer VWF chains. However, although the relative extension (i.e., normalized by *L*_VWF_) of the longer VWF is less compared with the shorter VWF, the physical extension of the longer VWF is larger than that of a shorter VWF.

**Figure 5:**
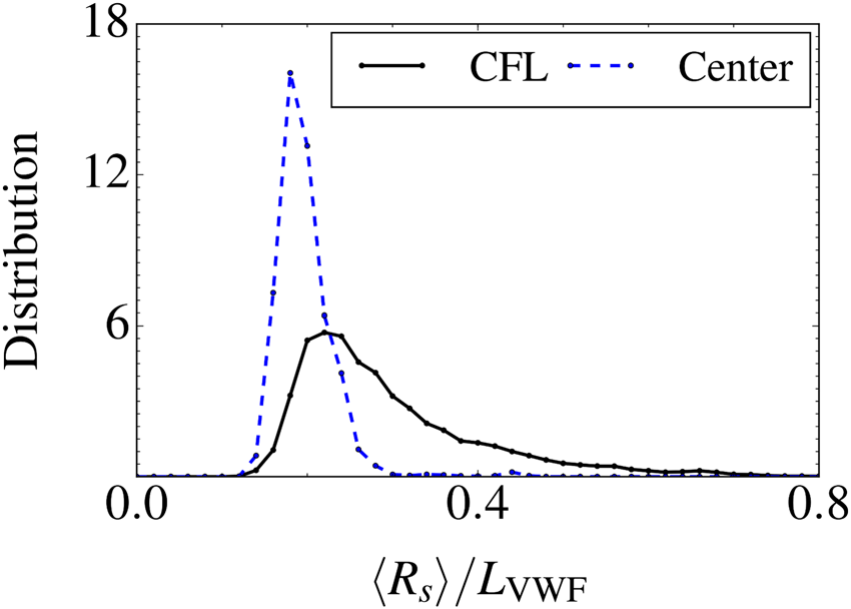
VWF extension within a channel cross-section. Distributions of VWF extension in the RBC-flow core and RBC-FL, showing that a significant stretching is achieved mainly in the RBC-FL. 2D simulation for the parameters *H*_*t*_ = 0.3 and ${\dot{\gamma }}^{\ast }\approx 29.7$ ($\bar{\dot{\gamma }}\approx 31$ s^−1^).

These results allow us to draw a qualitative picture for VWF behavior in blood flow. VWF remains primarily in a compact configuration within the RBC-flow core, which leads to its efficient margination into RBC-FL. However, VWF is able to stretch near the wall due to high shear rates and the soft confinement between RBCs and the wall. To confirm this proposition, we plot in Fig. 5 the distributions of VWF extension in the RBC core and RBC-FL. Even though some moderate stretching in the RBC core is possible, the largest VWF extension clearly occurs within the RBC-FL. This indicates that the adhesive interactions of VWF with vessel walls or other blood components should be mainly expected in the RBC-FL.

### VWF adhesion

As already mentioned, VWF margination and stretching are two necessary pre-conditions for adhesion such that their convolution characterizes the potential for VWF adhesion. Even though we do not study VWF adhesion directly in simulations, we have performed data analysis in such a way that we can estimate the potential-adhesion probability Ψ for various conditions. This estimation is based on two conditions: (i) a monomer in VWF chain has to be close enough to the channel wall in order to be able to adhere and (ii) this monomer has to be active for adhesion. The second condition mimics the unfolding of VWF factor in flow such that hidden adhesive sites become exposed and the VWF adhesion becomes possible^1,20^. This condition is realized through the degree of local stretching of VWF in flow, see *Methods* for details. Then, the potential-adhesion probability is calculated as ${\rm{\Psi }}=({\sum }_{i}^{{N}_{time}}{N}_{act}^{i})/({N}_{tot}{N}_{time})$, where ${N}_{act}^{i}$ is the number of active VWF beads within the near-wall layer ${\delta }_{{\rm{adhes}}}$ at a time instance *i*, ${N}_{time}$ is the total number of time instances taken into account, and ${N}_{tot}$ is the total number of VWF beads in a simulation domain.

**Figure 6:**
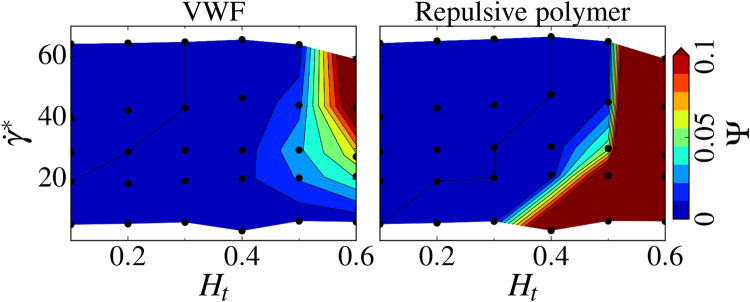
Potential-adhesion probability of VWF and a repulsive polymer. Potential-adhesion probability Ψ of VWF and a repulsive polymer as a function of *H*_*t*_ and shear rate ${\dot{\gamma }}^{\ast }$. The probability of potential adhesion is calculated based on two conditions: (i) a chain monomer has to be close enough to the channel wall (${\delta }_{{\rm{adhes}}} < 1\,{\rm{\mu }}{\rm{m}}$) and (ii) this monomer has to be activated for adhesion which is characterized by the degree of local stretching of the polymer chain (see *Methods* for details). Thus, Ψ is the adhesion probability defined as the average fraction of active polymer beads in close proximity to the walls. Both the VWF and repulsive polymer consist of 26 beads corresponding to the contour length of *L*_VWF_ = 15 μm. The black dots indicate the values of *H*_*t*_ and ${\dot{\gamma }}^{\ast }$ for which simulations have been performed. The color code ranges from blue (low probability) to red (high probability) and is acquired via interpolation.

Figure 6 compares diagrams for the potential-adhesion probabilities of VWF and a repulsive polymer. In both cases, adhesion at low *H*_*t*_ values is extremely limited independently of the shear rate due to poor margination, such that polymers remain far enough from the wall and cannot adhere. However, if *H*_*t*_ becomes high enough (${H}_{t} > 0.4$ in 2D corresponding to ${H}_{t} > 0.2$ in 3D) the adhesion becomes possible as the polymers marginate well. In this *H*_*t*_ range, VWF can adhere mainly at large enough shear rates, since at low shear rates VWF does not stretch enough to expose its adhesive sites. In contrast, the adhesion of a repulsive polymer is very much possible at low as well as high shear rates, because it can easily stretch independently of the shear rate, as seen in Fig. 2. This analysis demonstrates that VWF adhesion in blood flow is the convolution of its margination and stretching, and strongly depends on local blood-flow characteristics within the microcirculation.

Our simulations show that VWF or repulsive polymers can stretch within the RBC-FL and form a contact with the wall, while recent investigations^49,50^ suggest that in order to adhere, a VWF must remain in a compact form, since in a stretched configuration it will immediately be pushed away from the wall by the lift force^47^. The proposed mechanism for adhesion proceeds by first attaching a single monomer to the wall with a subsequent VWF elongation. The main difference between these studies^49,50^ and our simulations is that the previous studies have been performed for a simple shear flow without RBCs. However, the situation in blood flow is strongly affected by the presence of RBCs. In blood flow, VWF marginates and remains quasi-trapped in the RBC-FL between the wall and flowing RBCs. Thus, even in a partially stretched configuration, VWF does not immediately demarginate due to the interactions with flowing RBCs. Then, tumbling dynamics of a partially-stretched VWF within the RBC-FL facilitates a contact with the wall. Even though the adhesion mechanism proposed in refs^49,50^ should still be possible in blood flow, our simulations show that the other mechanism described above is dominating in blood flow.

**Figure 7:**
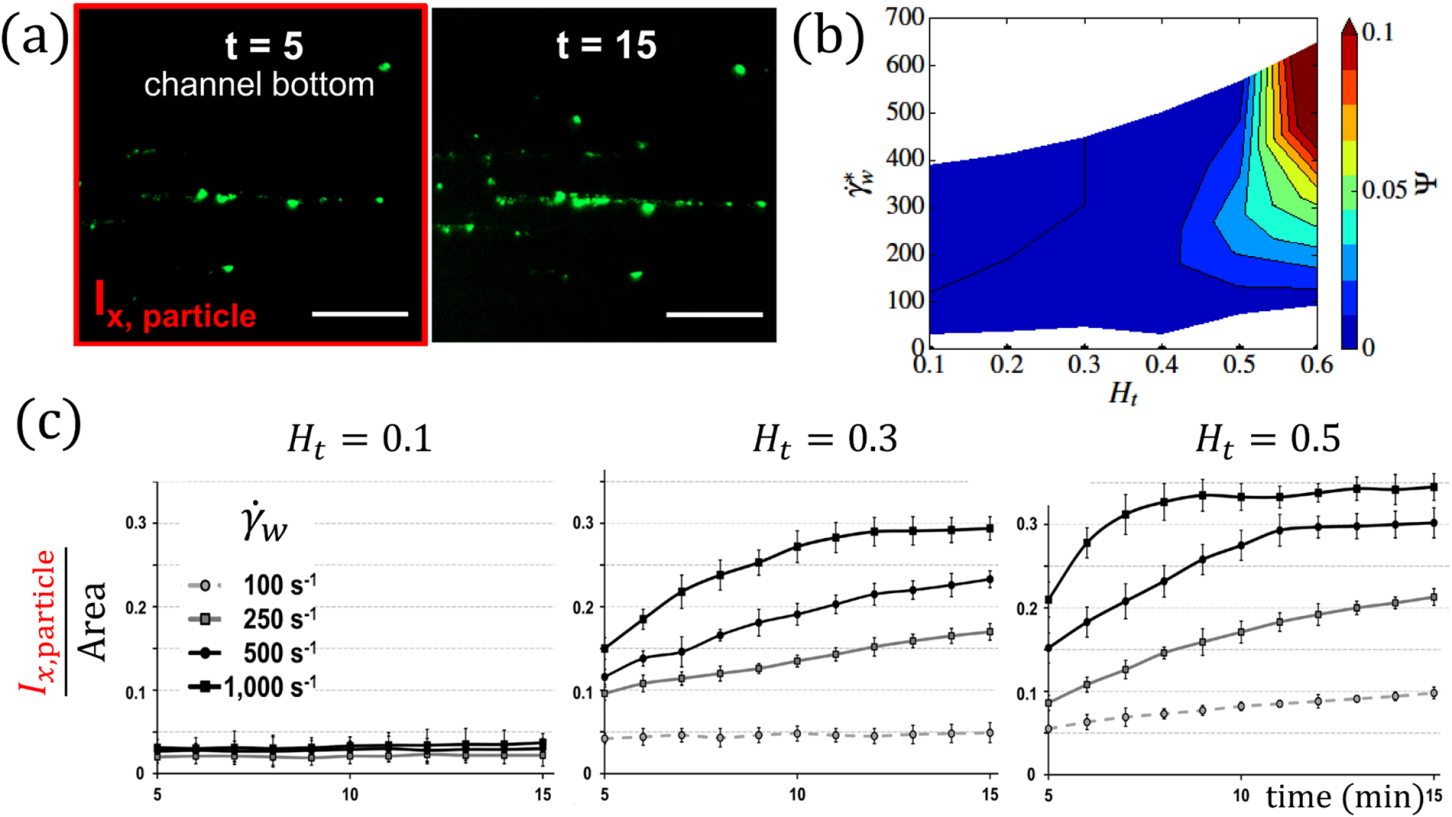
VWF adhesion in experiments. (**a**) VWF adhesion is monitored over time and represented by the intensity and area of adhered VWF spots at the channel bottom, as shown by the two successive images of the experiment for *H*_*t*_ = 0.5 at ${\dot{\gamma }}_{{\rm{w}}}=500$ *s* ^−1^. The isotropic background level of intensity, which characterizes VWF margination, is subtracted. (**b**) Potential-adhesion probability Ψ estimated from simulations as a function of *H*_*t*_ and ${\dot{\gamma }}_{{\rm{w}}}^{\ast }$ for *N* = 26 (*L*_VWF_ = 15 μm). It characterizes the probability of adhesion which is related to the slope of VWF-adhesion curves in experiments at short times before the adhesion saturation has been reached. (**c**) Adhesion of VWF to collagen in microfluidic channels for various wall shearrates and hematocrits (*H*_*t*_ = 0.1, *H*_*t*_ = 0.3, *H*_*t*_ = 0.5). VWF adhesion is monitored over time. The error bars in the plots show the deviation of monitored data from four independent experiments.

To study VWF adhesion directly, we performed a set of microfluidic experiments, with the results presented in Fig. 7. VWF adhesion is quantified by measuring in time the intensity ${{\rm{I}}}_{x,\mathrm{particle}}$ of fluorescent spots at the bottom of the channel within an observation area, where the isotropic background intensity related to VWF margination is subtracted, see Fig. 7(a). For comparison, Fig. 7(b) presents the potential-adhesion probability of VWF estimated from simulations as a function of *H*_*t*_ and wall shear rate ${\dot{\gamma }}_{{\rm{w}}}^{\ast }$, which can be interpreted as an adhesion rate of a VWF molecule if we multiply it by the rate of forming a bond between VWF monomer and a surface. Thus, the potential-adhesion probability should be proportional to the adhesion rate of a VWF molecule and characterizes qualitatively the degree of accumulation of adhered VWF fluorescent spots followed in time in experiments.

The experimental results of VWF adhesion, presented in Fig. 7(c), display three principal behaviors for short times ($\lesssim 10$ min after perfusion with VWF-rich medium): no adhesion, weak adhesion, and strong adhesion. For longer times ($\gtrsim 10$ min), adhesion levels saturate and are no longer indicative of the adhesion probability.

For low hematocrit, *H*_*t*_ = 0.1, VWF adhesion remains absent, independent of the applied shear rates, due to weak VWF margination (Fig. 2). In fact, the two highest shear rates in Fig. 7(c) should be large enough to achieve the VWF extension needed for adhesion, but VWF chains flow too far away from the wall to bind. For moderate hematocrit, *H*_*t*_ = 0.3, VWF adhesion displays a pronounced dependence on shear rate. Here, the volume fraction of RBCs is large enough for pronounced margination, and therefore VWF adhesion becomes possible when VWF chains are able to stretch. For the lowest shear rate, ${\dot{\gamma }}_{w}=100$ *s* ^−1^, no significant adhesion occurs in the experiments, even though the results in Figs 2 and 3 indicate that there should be considerable VWF margination (*H*_*t*_ = 0.3 in 3D corresponds to ${H}_{t}\approx 0.5$ in 2D). Here, VWF extension is too weak,which is fully consistent with the potential-adhesion probability in Fig. 7(b). In contrast, at the highest wall shear rate, ${\dot{\gamma }}_{w}=1,000$ *s* ^−1^, a substantial increase in VWF adhesion is observed due to the enhanced VWF ability to stretch. Even though margination decreases with increasing shear rate (see Figs 2 and 4), VWF stretching over-compensates the reduced margination (see Fig. 7(b)). Finally, for high hematocrit, *H*_*t*_ = 0.5, VWF adhesion is pronounced and a large adhesion rate (i.e., slope of intensity versus time) is observed, again qualitatively consistent with the potential-adhesion probabilities in Fig. 7(b). Here, both margination and stretching are significant and favor strong adhesion. Interestingly, even for low shear rate, ${\dot{\gamma }}_{w}=100$ *s* ^−1^, some moderate adhesion is detected, indicating that VWF exhibits some stretching due to the confinement effect, since at *H*_*t*_ = 0.5 the thickness of RBC-FL is comparable with the VWF size.

## Discussion

The results presented above illustrate the behavior of large multimeric VWFs in blood flow. Within the flow core mainly consisting of RBCs, VWF maintains its compact shape and is not subject to significant stretching. This property not only prevents unwanted adhesion of VWF to blood cells, but also facilitates efficient VWF margination. Here, the large size of the VWF protein, which implies a characteristic diameter of a few microns, is also important, since this size seems to be optimal for margination^30,31,37^, as discussed in more detail below. In contrast, the results for a repulsive polymer without internal attractive interactions clearly show poor margination in comparison to VWF (see Fig. 2). After margination, VWF stretches near the wall, which may result in its wall adhesion. Thus, both margination and stretching of VWF need to directly precede adhesion, and VWF behavior strongly depends on hematocrit and shear rate. Adhesion of VWF at a low *H*_*t*_ is limited by margination independently of whether VWF stretches or not (see Fig. 7). Furthermore, at low wall shear rates, VWF stretching can be a limiting factor for adhesion, even if VWF margination is significant. However, when both conditions are fulfilled, VWF adhesion becomes possible, as seen in Fig. 7.

The compact shape of VWF allows the comparison of its margination properties with those of rigid nano- and micro-particles. Recent investigations^30,31,37^ have shown that micron-sized particles possess much better margination properties than their sub-micron counterparts. This implies that VWF chains with a relatively short length ($\lesssim$3–5 μm), such that they have a sub-micron diameter, are expected not to marginate as well as larger VWFs. Our results in Figs 2 and 4 show that VWFs with a length larger than about 12–13 μm should possess best margination properties in blood flow. Furthermore, the distribution of very short VWF multimers (of only a few dimers) in blood is expected to be similar to the blood-plasma distribution, since very small particles ($\lesssim 200$ nm) have a nearly homogeneous distribution within the plasma^31^.

Another interesting result of our study is that VWF adhesion is possible even for very low shear rates at high *H*_*t*_. The prediction of critical shear rate required for VWF stretching in free flow in the absence of RBCs corresponds to several thousand *s* ^−1^ ^21^; however, at *H*_*t*_ = 0.5 in Fig. 7, VWF adhesion was observed at shear rates ten times smaller than this value. This is due to the confinement effect of VWF in the RBC-FL, which leads to an enhanced stretching of VWF and possible adhesion. Such spontaneous adhesion might be of importance in tumor microvasculature, where local hematocrit is often elevated due to plasma leakage through endothelial fenestrations^51^. Furthermore, VWF is also able to stretch at relatively low shear rates after a single adhesion point has been formed^49,50^, facilitating further adhesion interactions.

The comparison between VWF and a repulsive polymer shows that the strength of internal associations (attractive interactions between monomers) within VWF defines not only its margination properties, but also its stretching behavior, where a repulsive polymer is able to stretch at much lower shear rates than VWF. Certain mutations of VWF may affect its physical properties, resulting in the reduction or intensification of the internal associations within VWF. Such changes are expected to strongly affect VWF stretching and margination behavior as was illustrated in Fig. 2. In addition, stretching of VWF in blood flow is also correlated with the activity of ADAMTS13 protease, which cuts VWF at an open cleavage site within the A2 domain^8^. Our results indicate that VWF cleavage should mainly occur in the RBC-FL in microcirculatory locations with high enough *H*_*t*_, where the cleavage site of VWF is exposed to ADAMTS13 ^1,11^.

In conclusion, VWF exhibits an intricate behavior in blood flow, which depends on a number of conditions including flow rate, hematocrit, VWF length, and intra-protein attraction. The performed simulations provide a theoretical understanding for the complex dynamics of VWF in blood flow, which is entirely consistent with our experimental observations of VWF adhesion in microfluidics. These results are important for the understanding of VWF distribution and adhesion in the microvasculature in health and disease.

## Methods

### Simulation method

Simulations are based on the dissipative particle dynamics (DPD) method^52^ in 2D and the smoothed DPD method^53,54^ in 3D, which are both particle-based hydrodynamics simulation approaches. The simulated systems are represented by a collection of point particles, which interact through pairwise soft potentials and move according to the Newton’s second law of motion. In both approaches, particles interact by three different forces: conservative *F* ^*C*^, dissipative *F* ^*D*^, and random *F* ^*R*^. The conservative force controls fluid compressibility, while the pair of dissipative and random forces defines a local thermostat in order to keep the system at an equilibrium temperature. In the SDPD method, the forces are derived by a discretization of the Navier-Stokes equation, while the imposition of thermal fluctuations is analogous to that in DPD^53^. In contrast to the DPD method, SDPD allows the direct input of the dynamic viscosity of a fluid as well as its equation of state. Thus, the fluid compressibility can be controlled much better in SDPD than in DPD. Equations of motion of the system are integrated using the velocity-Verlet algorithm. Table S1 presents the fluid parameters used in the 2D (DPD) and 3D (SDPD) simulations.

### Simulation setup and conditions

The simulation setup consists of a single channel of a cylindrical shape in 3D with a diameter *W* = 20 μm and a length $L=\mathrm{12.3\ }{D}_{r}$. In 2D, we use slit geometry with a width *W* = 20 μm and a length $L=28.6{D}_{r}$. The channel is filled with fluid particles according to the particle densities in Table S1 and with suspended VWFs and RBCs. The number of RBCs is determined by the selected hematocrit, which corresponds to the volume fraction of RBCs in 3D and to the area fraction of RBCs in 2D. For both 3D and 2D simulations the number of VWFs is ${N}_{{\rm{V}}WF}=6$. The volume fraction of VWFs remains very small in all simulations such that the effect of concentration on its behavior can be neglected.

In the flow direction, periodic boundary conditions (BCs) were imposed, while in the other directions the suspension was confined by walls. The walls are modeled by frozen fluid particles with the same structure as the fluid. Thus, the interactions of fluid particles with wall particles are the same as fluid-fluid interactions, and the interactions of structures with the wall are identical to those with a suspending fluid. To prevent wall penetration, fluid particles as well as vertices of RBCs and beads of VWFs are subject to reflection at the fluid-solid interface. We employed bounce-back reflections, because they provide a better approximation for the no-slip boundary conditions than specular reflection of particles. To ensure that no-slip BCs are strictly satisfied on the walls, we also add a tangential adaptive shear force^54^ which acts on the fluid particles in a near-wall layer.

### RBC models

**Figure 8:**
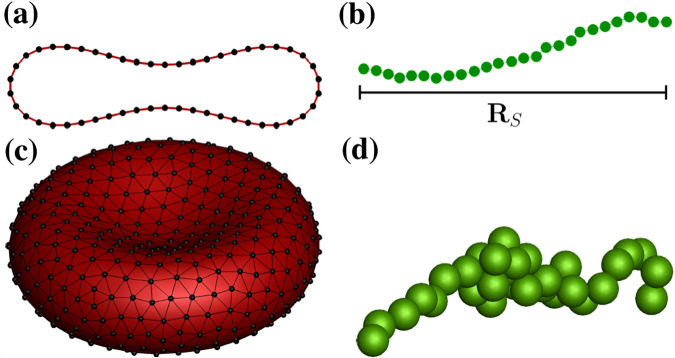
Simulation models. Models of (**a**,**c**) RBCs and (**b**,**d**) VWFs in 2D (top) and 3D (bottom). For the RBCs, the vertices are marked by black dots and the triangulated network is drawn in black. The extension of polymer, *R*_*s*_, in the flow direction is shown in (**b**).

In 3D, the membranes of RBCs are modeled by a triangulated network of ${N}_{s}=\mathrm{3(}{N}_{v}-\mathrm{2)}$ springs^55–58^ with *N*_*v*_ vertices, see Fig. 8(c). The potential energy of a membrane is defined as ${U}_{{\rm{m}}em}={U}_{{\rm{s}}}+{U}_{{\rm{b}}}+{U}_{{\rm{A}}}+{U}_{{\rm{V}}}$, where *U*_s_ is the spring’s elastic energy, *U*_b_ is the bending energy, and *U*_A_ and *U*_V_ correspond to the area and volume conservation constraints. The spring energy mimics the elasticity of a membrane. The bending energy represents the bending resistance of a membrane, while the area and volume energies enforce area-incompressibility of a membrane and incompressibility of the inner cytosol, respectively. Detailed description of these potentials can be found in refs^58,59^.

We employ a stress-free model for the membrane^58^ such that each spring has its own equilibrium length *l*_0_ set to the spring length of an initially triangulated discocyte surface. For the RBCs with ${N}_{v}=500$, we set ${l}_{{\rm{m}}}/{l}_{0}=2.2$ for all springs, where *l*_m_ is the maximum spring extension. All model notations here are the same as in ref.^58^. Spring parameters can be related to the Young’s modulus *Y* of a membrane^58^, and we assume that $Y=18.9\times {10}^{-6}\,N/m$ for the RBCs^58^. The spontaneous angle *θ*_0_ between two adjacent faces is set to zero. The bending constant *k*_b_ is related to the macroscopic bending rigidity ${\kappa }_{r}$ as ${k}_{{\rm{b}}}=2{\kappa }_{r}/\sqrt{3}$, and a value for healthy RBCs is ${\kappa }_{r}=70{k}_{B}T$ ^58^.

A unit length *l* in simulations corresponds to 1 μm in physical units. We define the cell effective diameter as ${D}_{r}=\sqrt{{A}_{0}/\pi }$ with the RBC area ${A}_{0}=133{l}^{2}$ in all simulations, which corresponds to ${A}_{0}=133$ μm^2^. The RBC volume is set to ${V}_{0}=92.5$ μm^3^. Furthermore, we set the local area constraint coefficient to ${k}_{d}=42250{k}_{B}T/{D}_{r}^{2}$, the global area constraint coefficient to ${k}_{a}/{k}_{d}=49$, and the volume constraint coefficient to ${k}_{v}{D}_{r}/{k}_{d}=325$.

In the 2D simulations, the RBCs are modeled as bead-spring chain rings^60^ using *N*_*v*_ particles connected by ${N}_{s}={N}_{v}$ springs, see Fig. 8(a). For the RBCs, ${N}_{v}=50$ and the spring potential is the same as in ref.^60^. The bending rigidity is imposed for every angle between two neighboring springs with the spontaneous angle ${\theta }_{0}=0$ and ${k}_{b}=50{k}_{B}T$. The area constraint of a membrane has the same expression as in ref.^60^. The effective RBC diameter is defined here as ${D}_{r}={L}_{0}/\pi$ with the RBC circumference ${L}_{0}=19.22$ μm. Furthermore, the area constraint coefficient is set to ${k}_{a}=17640{k}_{B}T/{D}_{r}^{2}$.

Coupling between the fluid and suspended structures such as RBCs and VWF is achieved through viscous friction^58^ between the vertices/monomers and the surrounding fluid particles, which is implemented via the DPD interactions *F* ^*D*^ and *F* ^*R*^ for both 2D and 3D simulations. The strength of the dissipative force *F* ^*D*^ for the interaction between a fluid particle and a membrane vertex/monomer is computed such that no-slip BCs are ensured on the surface of RBCs or along VWF chain. Aggregation interactions between RBCs are not included in this study, because their effect is important mainly at shear rates below about 10 *s* ^−1^ in simple shear flow^61^, which are too low for VWF stretching and therefore, are not considered here.

### VWF model

As proposed in refs^22,42^ the VWF is modeled as a self-avoiding bead-spring chain with intramolecular attractive interactions and a monomer radius *a*_m_, see Fig. 8(b,d). The resultant potential for polymer beads is the superposition of a harmonic bond potential, with stiffness *k*_*s*_ and equilibrium bond length ${l}_{{\rm{b}}}=2{a}_{{\rm{m}}}$, and an attractive Lennard-Jones (LJ) pairwise potential with $\sigma ={l}_{{\rm{b}}}$ and $\varepsilon =4{k}_{{\rm{B}}}T$; this value is a result of the calibration of a critical shear rate for polymer stretching in 3D against experimental data in ref.^21^ shown in Supplementary Fig. S1. In 2D, the depth of the LJ potential is kept the same. The VWF model includes the attractive part of LJ interaction while the repulsive polymer model (e.g., self-avoiding polymer) excludes the attractive part. The equilibrium bond length is set to 0.6 μm in 2D and 0.5 μm in 3D simulations. The harmonic bond stiffness is ${k}_{{\rm{s}}}=400{k}_{{\rm{B}}}T{a}_{{\rm{m}}}^{-2}$ in 2D and ${k}_{{\rm{s}}}=3125{k}_{{\rm{B}}}T{a}_{{\rm{m}}}^{-2}$ in 3D in all presented simulations. In order to prevent overlap between RBCs and VWFs, we also employ the LJ potential only with its repulsive part.

In order to estimate the potential-adhesion probability **Ψ** of VWF, we employ two conditions: (i) a chain monomer has to be close enough to the channel wall and (ii) this monomer has be activated for adhesion which is characterized by the degree of local stretching of the polymer chain. The adhesive distance to the wall is assumed to be ${\delta }_{{\rm{adhes}}}=\mathrm{1\ }{\rm{\mu }}m$, and VWF polymer bead activity depends on how the polymer is stretched locally. In particular, a VWF polymer bead is considered to be active if it sees no more than its neighbor bound beads inside a threshold radius *R*_thres_ and if its bonds make up an angle more than a threshold angle *θ*_thres_ (this condition is not considered for the end beads). Consequently, the locally stretched VWF portion can become active while the collapsed portion remains inactive. The threshold criteria are chosen as *R*_thres_ = 1.2*l*_b_ and ${\theta }_{{\rm{thres}}}=130$ which provide satisfactory activity based on VWF configuration. As a result, ${\rm{\Psi }}=({\sum }_{i}^{{N}_{time}}{N}_{act}^{i})/({N}_{tot}{N}_{time})$, where ${N}_{act}^{i}$ is the number of active beads within a layer *δ*_adhes_, *N*_*time*_ is the total number of time instances considered, and *N*_*tot*_ is the total number of VWF beads in the simulation domain.

### Experimental setup and analysis

For microfluidic experiments, we use a pneumatically driven channel system (BioFlux, San Francisco, CA, USA), see Fig. 1(b). Its pressure interface connects a high precision electropneumatic pump to the well plates to initiate a controlled flow rate with a nominal shear rate precision of 36 s^−1^. The channels with a size of 350 × 75 μm are coated with collagen type I (C7661, Sigma Aldrich, St. Louis, MO, USA) in a concentration of 0.01% for one hour at 37 °C. The microfluidic channel system is mounted onto an inverted microscope (Zeiss Axio Observer Z.1, Zeiss AG, Oberkochen, Germany). Image acquisition is performed using a CCD camera (AxioCam MrM) and ZEN software (both Zeiss AG, Jena, Germany).

For microfluidic measurements, perfusion media with different hematocrit levels containing EGFP-marked VWF are perfused through the collagen-coated channels at distinct wall shear rates. After five minutes of continuous perfusion, margination of VWF is measured through the intensity of fluorescently labeled VWF near the channel bottom (*I*_x_) in an area free of adhered VWF particles, and is normalized by the wall autofluorescence intensity *I*_w_, which remained constant throughout all performed experiments. To analyze the impact of hematocrit on adhesion of marginated VWF to the collagen-coated channel bottom, the fluorescence intensity of adhered VWF particles is quantified. Under distinct shear rates of 100 s^−1^, 250 s^−1^, 500 s^−1^, and 1,000 s^−1^, the surface coverage of adhered VWF particles per observation area is monitored in time. For image analysis, we use ZEN software (Zeiss AG, Jena, Germany) and the open-source software ImageJ (V. 1.46r, National Institute of Health, Bethesda, MD, USA). Mean data from experiments are given with standard deviation (SD).

### Experimental materials

For recombinant VWF expression, we use HEK293 cells. Cells are cultured in Dulbecco Modified Eagle Medium (DMEM, Invitrogen, Karlsruhe, Germany) with 10% fetal bovine serum and 1% penicillin/streptavidin at 37 °C. Then, cells are transfected with Lipofectamine 2,000 (Invitrogen, Karlsruhe, Germany) and VWF-plasmid-constructs in vector pIRESneo2. Recombinant expression of VWF is performed as follows. In brief, HEK293 cells stably express VWF. Samples of the supernatant are taken, centrifuged (5 min at 270 g, 4 °C), concentrated with Amicon Ultrafree-15, and VWF concentration is determined by VWF:Ag-ELISA. VWF is fluorescently marked by EGFP via a His-tag; EGFP-VWF has been provided by the group of R. Schneppenheim (University Medical Center Eppendorf, Hamburg, Germany) and added to the perfusion media in a concentration of 20 g/ml. Blood has been collected into citrated vacuum tubes from healthy volunteers after informed consent. To reach the indicated levels of hematocrit, RBCs are isolated from blood samples, washed and supplemented with HEPES buffered Ringer solution (HBRS) containing (in mmol/L): 140 NaCl, 5 KCl, 1 MgCl, 1 CaCl2, 5 glucose, 10 HEPES (N-2-hydroxyethylpiperazin-N0–2-ethanesulfonic acid) adjusted to pH 7.4. This study has been conducted in conformity with the Declaration of Helsinki and with the International Conference on Harmonisation of Technical Requirements for Registration of Pharmaceuticals for Human Use (ICH) Guidelines available at http://www.ich.org. It has been approved by the Ethics Committee II of the Heidelberg University (Mannheim, Germany).

## Electronic supplementary material

Supplementary Information

Movie S1

Movie S2

Movie S3

Movie S4

Movie S5

Movie S6
