# Supplementary material for: Margination and stretching of von Willebrand factor in the blood stream enable adhesion
Source: Sci Rep. 2017 Oct 27;7:14278. doi: 10.1038/s41598-017-14346-4 (PMC5660260; doi:10.1038/s41598-017-14346-4)
Supplement: Supplementary file 1 — Supplementary Information [file 41598_2017_14346_MOESM1_ESM.pdf]

SUPPLEMENTARY MATERIAL for

Margination and stretching of von Willebrand factor in the blood stream enable adhesion

Kathrin Rack, Volker Huck, Masoud Hoore, Dmitry A. Fedosov, Stefan W. Schneider, and Gerhard Gompper

Supplementary methods

The dissipative particle dynamics (DPD) [1] and smoothed DPD (SDPD) [2] with angular momentum conservation [3] are employed for 2D and 3D simulations, respectively. In contrast to the DPD method, SDPD allows us to input directly the dynamic viscosity of a fluid as well as its equation of state. Thus, the fluid compressibility can be controlled much better in SDPD than in DPD. Equations of motion of the system are integrated using the velocity-Verlet algorithm. Supplementary Table S1 presents the fluid parameters used in the 2D (DPD) and 3D (SDPD) simulations. The notations for 2D and 3D cases are the same as in Refs. [4] and [3], respectively.

DPD[1] (2D)								
	m	a	γ	r_c	s	n	k_BT	η
$\dot{\gamma}^* \leq 7$	1	40	10	1.5	0.3	5	1	72.2
$\dot{\gamma}^* > 7$	1	40	20	1.5	0.3	5	1	144.4
**SDPD[2] (3D)**								
p_0	ρ_0	α	b	r_c	η_0	n	k_BT	η
100	3.0	7	80	1.5	100.0	3	0.4	107.6
[1] Notations as in Ref. [4]						[2] Notations as in Ref. [3]		

Supplementary Table S1. Simulation parameters. DPD parameters for 2D simulations: m is the mass of a fluid particle, a and γ are the conservative and dissipative force coefficients, respectively. r_c is the cutoff radius, s is an exponent for the random-force weight function, n is the number density of fluid particles, k_BT is an energy unit with k_B being the Boltzmann constant and T is temperature, and η is the fluid's dynamic viscosity. The notations are the same as in Ref. [4]. SDPD fluid parameters used in 3D simulations: p_0, ρ_0, α, and b define the pressure equation of state. Furthermore, r_c is the cutoff radius, η_0 is the desired dynamic viscosity, n is the number density of the fluid, k_BT is the energy unit, and η is the measured (real) fluid viscosity. The notations are the same as in Ref. [3].

Supplementary figures

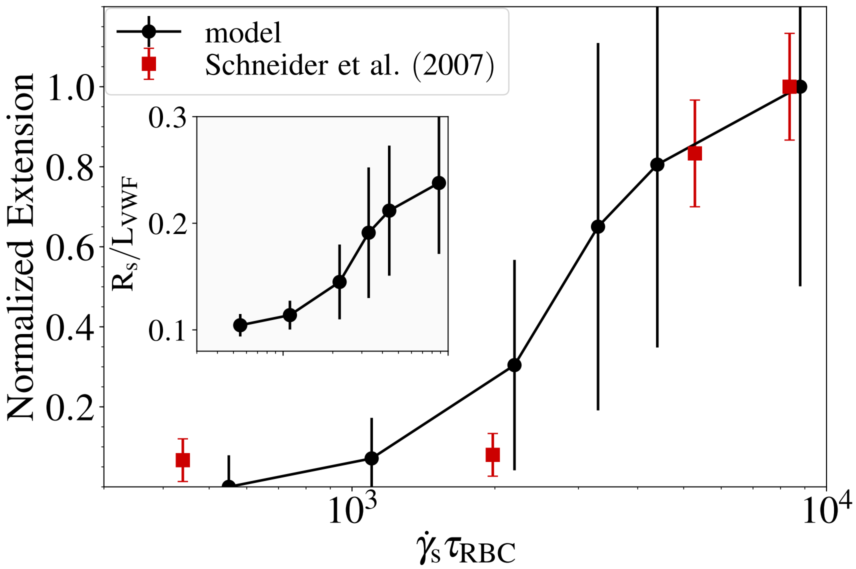

Supplementary Figure S1. Calibration of the 3D VWF model against experiments. Comparison of simulated VWF stretching in simple shear flow with experiments [5], where the critical shear rate for polymer stretching in the model is adjusted by changing the attractive strength of LJ potential. The model employs the LJ potential with $\sigma = l_\mathrm{b}$ and $\epsilon = 4k_\mathrm{B}T$. The maximum extension point is normalized to unity for both simulation and experimental data. $\dot{\gamma}_s$ is the shear rate, which is normalized by the characteristic relaxation time of a RBC τ_RBC ($\tau_\mathrm{RBC} \approx 1.1$ s) in order to properly relate the time scale of VWF stretching and τ_RBC. Inset shows the simulated extension normalized by VWF's contour length L_VWF.

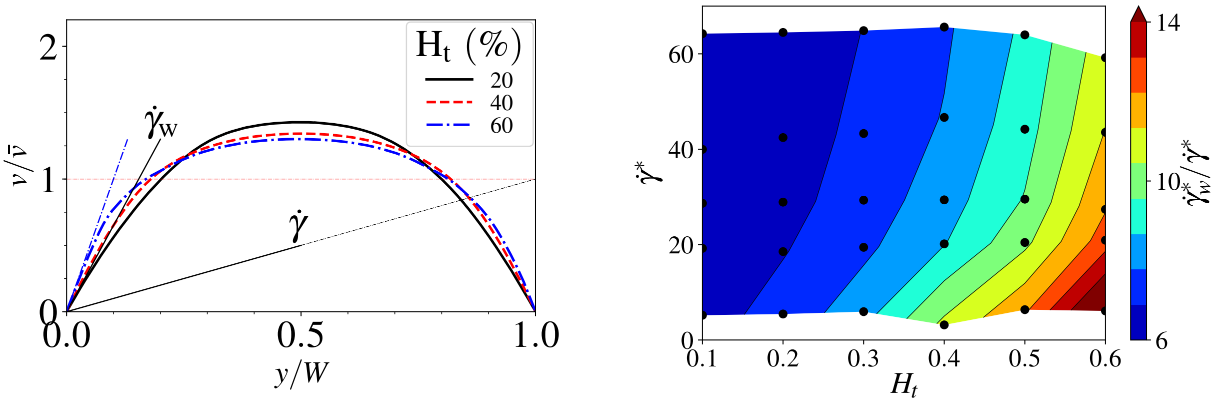

Supplementary Figure S2. Relation between the wall-shear rate and the average shear rate. (left) Flow velocity profiles for different hematocrit values at $\dot{\gamma}^* = 30$. Wall shear rates $\dot{\gamma}_\mathrm{w}$ for different H_t are indicated by tangent lines to the velocity profiles near the wall. The average shear rate $\dot{\gamma}$ is indicated by the solid line. **(right)** Diagram showing the ratio between the wall-shear rate and the average shear rate in 2D for various H_t values and flow conditions. The black dots indicate the values of H_t and $\dot{\gamma}^*$ for which simulations have been performed.

Supplementary movies

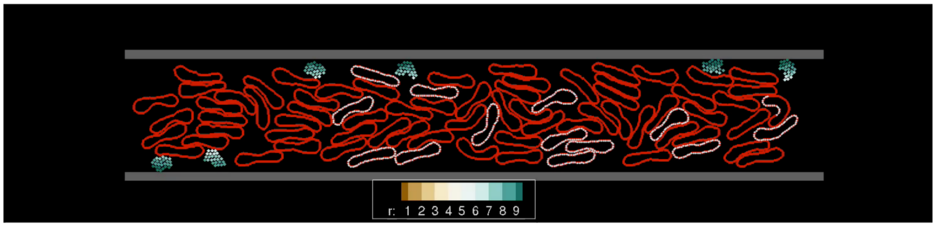

Movie S1: 2D blood flow simulation with VWF ($N = 26$) for small shear rate $\dot{\gamma}^* = 6$ and $H_t = 0.5$. Most of RBCs are drawn in red, while a few of them are colored white to follow better the flow. VWF monomers have a range of colors which correspond to their position in the channel (r ranges from 0 in the center to $W/2$ at the walls).

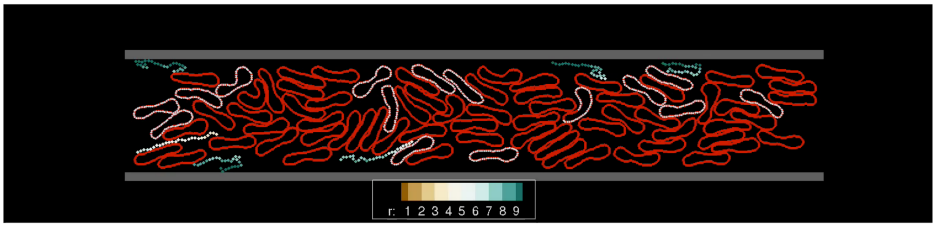

Movie S2: 2D blood flow simulation with repulsive polymers ($N = 26$) for small shear rate $\dot{\gamma}^* = 6$ and $H_t = 0.5$. Most of RBCs are drawn in red, while a few of them are colored white to follow better the flow. Polymer monomers have a range of colors which correspond to their position in the channel (r ranges from 0 in the center to $W/2$ at the walls).

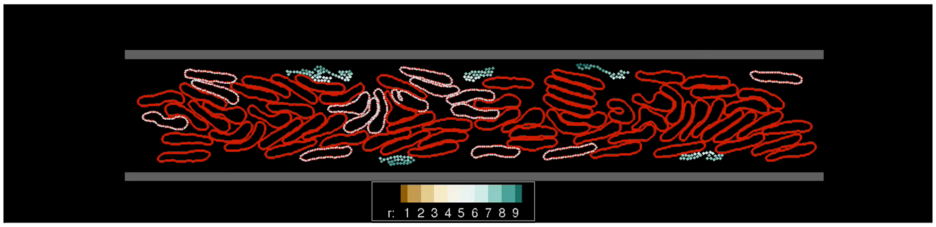

Movie S3: 2D blood flow simulation with VWF ($N = 26$) for high shear rate $\dot{\gamma}^* = 63$ and $H_t = 0.5$. Most of RBCs are drawn in red, while a few of them are colored white to follow better the flow. VWF monomers have a range of colors which correspond to their position in the channel (r ranges from 0 in the center to $W/2$ at the walls).

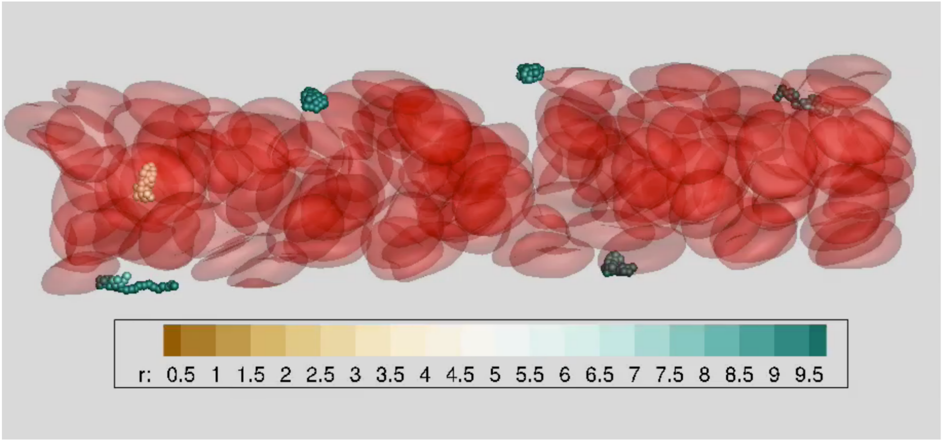

Movie S4: 3D blood flow simulation with VWF ($N = 26$) for $\dot{\gamma}^* = 20$ and $H_t = 0.5$. RBCs are drawn in red and partially transparent to see better the motion of VWF. VWF monomers have a range of colors which correspond to their position in the cylindrical channel (r ranges from 0 in the center to $W/2$ at the walls).

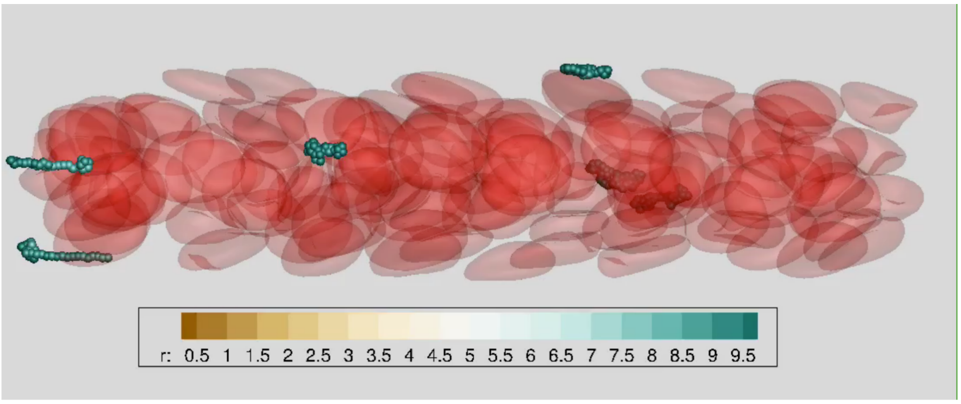

Movie S5: 3D blood flow simulation with VWF ($N = 26$) for $\dot{\gamma}^* = 60$ and $H_t = 0.5$. RBCs are drawn in red and partially transparent to see better the motion of VWF. VWF monomers have a range of colors which correspond to their position in the cylindrical channel (r ranges from 0 in the center to $W/2$ at the walls).

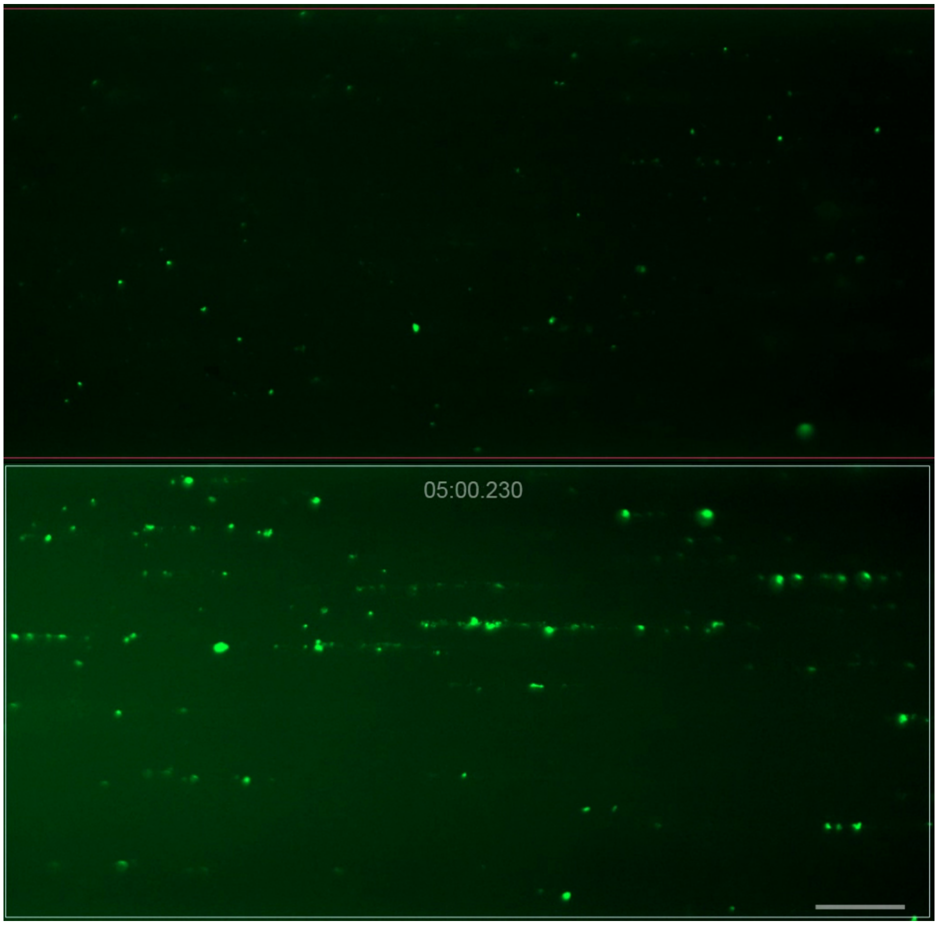

Movie S6: Experimental video of monitoring the adhesion of fluorescently labeled VWF near the channel bottom in time. The top part of the video corresponds to $H_t = 0.1$ and the bottom part of the movie to $H_t = 0.5$. The recorded time is in minutes. Scale bar corresponds to 50 μm.

Supplementary references

[1] Hoogerbrugge, P. J. & Koelman, J. M. V. A. Simulating microscopic hydrodynamic phenomena with dissipative particle dynamics. *Europhys. Lett.* **19**, 155–160 (1992).

[2] Español, P. & Revenga, M. Smoothed dissipative particle dynamics. *Phys. Rev. E* **67**, 026705 (2003).

[3] Müller, K., Fedosov, D. A. & Gompper, G. Smoothed dissipative particle dynamics with angular momentum conservation. *J. Comp. Phys.* **281**, 301–315 (2015).

[4] Müller, K., Fedosov, D. A. & Gompper, G. Margination of micro- and nano-particles in blood flow and its effect on drug delivery. *Sci. Rep.* **4**, 4871 (2014).

[5] Schneider, S. W. *et al.* Shear-induced unfolding triggers adhesion of von Willebrand factor fibers. *Proc. Natl. Acad. Sci. USA* **104**, 7899–7903 (2007).
